# Beyond the “Choosing wisely”: a possible attempt

**DOI:** 10.1186/s13052-016-0265-4

**Published:** 2016-05-28

**Authors:** Roberto Bernardini, Giampaolo Ricci, Francesca Cipriani, Flavio Civitelli, Luciana Indinnimeo, Domenico Minasi, Luigi Terracciano, Marzia Duse

**Affiliations:** Pediatric Unit, San Giuseppe Hospital, Empoli, Italy; Department of Medical and Surgical Sciences, Pediatric Unit, University of Bologna, S.Orsola-Malpighi Hospital, Pad 16, via Massarenti 11, 40138 Bologna, Italy; Pediatric Unit, Usl Sud Est Toscana, Montepulciano, Siena Italy; Department of Pediatrics and Child Neuropsychiatry, Policlinico Umberto I, Sapienza University of Rome, Rome, Italy; Pediatric Unit, Hospital of Polistena, Reggio Calabria, Italy; Pediatric Primary Care, National Pediatric Health Care System, Milan, Italy

**Keywords:** Allergy, Appropriateness, Certification, Choosing wisely, Health care resources, Procedures

## Abstract

Since the fundamental principles of the medical profession were clearly defined in a physician charter in 2002, special considerations have been expressed about the adequate distribution of health care resources taking in account the individual patient needs to optimize the health care service. The correct application of procedures represents a key point in order to reach the appropriateness of care, that means to avoid unnecessary or inappropriate procedures as well as the underutilization of the necessary procedures. In this context, the Choosing wisely campaign have been widely used and disclosed and even the Italian Society of Pediatric Allergology and Immunology - SIAIP has been working to make recommendations in order to ensure the appropriateness of care in the field of allergy and optimize the use of health care resources.

In 2002 the fundamental principles of the medical profession were clearly defined in a physician charter simultaneously published in *Annals of Internal Medicine* [1] and in *The Lancet* [2] and listed as follows: a) the interest of the patient; b) the autonomy of the patient; c) the social justice including the fair distribution of health care resources. The last item arises from the consideration that health care system is based on limited resources and hence physicians must take the responsibility for appropriate allocation of resources. This is a personal duty of a single physician but overall the duty of politicians who decide the allotment of health funds. It is necessary to meet the individual patient needs despite limited clinical activities.

In this way, it is crucial to detect the correct application of medical procedures in order to avoid unnecessary or inappropriate procedures as well as the underutilization of the necessary procedures: therefore, it is mandatory in our opinion to define the appropriateness of care. The first attempt to develop unequivocal criteria for determining the appropriateness of care has been proposed about 3 decades ago according to the RAND/University of California Los Angeles Appropriateness Method (RUAM) [3, 4]. The RUAM is an integrated process where evidences from scientific literature, in particular Evidence Based Medicine (EBM), are joined with the judgment of experts. EBM is unable by itself to support the decision in the majority of health problems and experience of different experts is needed to evaluate the criteria of appropriateness (more health benefit than harm) and of inappropriateness (health risk is likely to go beyond health benefit). The RUAM approach evaluated the appropriateness of the most frequent procedures especially in surgical field (i.e. coronary artery bypass, hysterectomy, prostatectomy) and invasive tecniques (i.e. colonscopy, endoscopy); however, even if many RUAM criteria have been proposed and accepted around the world, only a small part of the health system may be take it in consideration.

In the last years, another way to improve appropriateness in health care has been raised and it has been published in 2010 on *Archives of Internal Medicine* by Deborah Grady and Rita Redberg [5]. In their Editorial named “Less is more” they affirmed that physicians in United States provide more care than is needed for several reasons: patients’ expectation (they consider testing and intervention with better care), saving time (physician spend less time prescribing a test than to explain to the patient why is better not to treat) and not least, defensive medicine. The conclusion are that probably less health care can result in a better health.

In the same year, Howard Brody proposed that specialty societies chose unnecessary tests and interventions: a top five list of commonly used tests or treatments without evidence of benefit [6].

In this context, the American Board of Internal Medicine Foundation and Consumer Reports gave beginning in 2012 with the “Choosing Wisely” [7] a campaign to identify unnecessarily services increasing health costs: tests, procedures, and therapies overused, inappropriately used and potentially harmful. The main specialty societies identified 5 behavior recommendations that could be incorporated into the clinical practice of primary care providers in Family Medicine, Internal Medicine and Pediatrics. For example, the top 5 recommendations proposed for the pediatric hospital medicine are: 1) Do not order chest radiographs in children with uncomplicated asthma or bronchiolitis; 2) Do not use systemic corticosteroids in children under 2 years of age with a lower respiratory tract infection; 3) Do not use bronchodilators in children with bronchiolitis; 4) Do not treat gastroesophageal reflux in infants routinely with acid suppression therapy; 5) Do not use continuous pulse oximetry routinely in children with acute respiratory illness unless they are on supplemental oxygen [8]. Each recommendation is followed by a brief exposition with the reference to the source or guidelines to which it refers. The impact of the Choosing wisely campaign may change in different Countries and each recommendation could have different importance and cost-saving impact.

The Italian Society of Pediatric Allergology and Immunology (SIAIP) has proposed to 10 different Committees to identify commonly procedures or treatments without scientific evidence of benefit for children care. After two revisions by experts outside the commission, 16 recommendation with inappropriate procedure have been chosen and published in 2014 [9] (the first five recommendations are reported in Table 1).

**Table 1:** Five Recommendations of the Italian Society of Pediatric Allergy and Immunology – SIAIP

1	Avoid contraindicating routinely vaccination in case of allergies.
	A history of allergies or mild allergic reactions are not contraindications to vaccination.
	Local and mild systemic reactions (redness of the injection site and/or fever) after vaccination reactions are common and do not contraindicate the administration of doses of vaccine in the future. Special precautions should be followed only in the case of persons who have presented serious systemic reactions with risk of life (severe dyspnea, stridor, cyanosis, mental status changes, hypotension). The presence of sensitization to egg protein is not a contraindication to vaccination against measles, mumps and rubella.
	Kelso et al. 2012 [11], Kelso et al. 2013 [12]
2	Avoid performing routinely allergy testing in children with acute urticaria.
	The diagnosis of acute urticaria is basically clinical and infections (in particular viral infections) account the far most common cause during childhood. Testing patients for allergies is indicated only when there is a close temporal relationship between food ingestion and the appearance of urticarial eruption: laboratory investigations are not indicated in first instance, it is appropriate to limit allergologic tests to the skin test (SPT) by using commercial extracts or fresh food (prick by prick).
	Zuberbier et al. 2009 [13], Capra et al. 2012 [14], Zuberbier et al. 2009 [15]
3	Avoid prescribing mucolytics in children with bronchial asthma.
	Inflammation, mucosal edema and mucus hypersecretion increase the narrowing of the bronchial lumen with the formation of mucus plugs that worsen bronchial obstruction in patients with asthma. Studies conducted on the effectiveness of mucolytics to treat asthma and its exacerbations have demonstrated their poor effectiveness and the possibility of dangerous side effects. The most important International guidelines (GINA, ATS, BTS) don’t include mucolytics in the “management” of children with bronchial asthma. Mucolytics agents are also contraindicated under 2 years of age due to the risk of a substantial deterioration of respiratory function for a difficult bronchial drainage.
	Balsamo et al. 2010 [16], Aliyali et al. 2010 [17], Linee guida GINA italiane 2013 [18]
4	Avoid prescribing routinely immunological tests in children with recurrent respiratory infections.
	Immunological and genetic investigations are not need when the child is suffering from undifferentiated common viral infections affecting the upper airways and when there is no family history of primary lung diseases or hereditary immunodeficiencies. The decision to perform tests should be based not only on the number of infections, but expecially on their severity, on the presence of unusual or opportunistic germs, on the protracted course and on the occurrence of infections beyond the age of primary socialization. Complete blood cell count and the dosage of immunoglobulins are considered first level tests, together with the sweat test in patients with recurrence of ear infections, bacterial sinusitis, bronchopneumonia or other invasive infections.
	Notarangelo 2009 [19], Brand et al. 2012 [20], Bousfiha et al. 2013 [21]
5	Avoid ruling out a food from the diet only for the positivity of skin prick tests and/or specific serum IgE.
	An accurate medical history is essential for the diagnosis of food allergy, in particular should be investigated a framework compliant with food allergy and a temporal relationship between the introduction of food and the appearance of symptoms. The presence of skin test (prick test) and/or positive serum specific IgE against foods indicates only a sensitization, condition that can be compatible with the intake of a food. For a correct diagnosis of food allergy an oral food challenge test must be provided (if the history and skin prick tests/specific serum IgE are not exhaustive for diagnosis).
	Boyce et al. [22], Burks et al. 2012 [23], Heinzerling et al. 2013 [24]

Tests, treatments and procedures at risk of inappropriateness in Italy that Physicians and Patients should talk about

The Choosing wisely campaign involved many specialty societies all around the world, in some cases inside the Slow Medicine, as in Italy where Slow Medicine launched the campaign “doing more does not mean doing better”.

The first aim, to reduce waste avoiding to perform commonly non-scientific procedures, is probably achieved by Choosing wisely, but this campaign still remains a spot action and must be implemented. The Italian Society of Pediatric Allergology and Immunology is trying to create a scientific and rationale model of health care: the aim of this project is to guarantee the appropriateness of the single procedures or treatments, a valid assignment both to basic Allergology service and to second or tertiary service.

After an extended evaluation by the Board, the Society decided to identify a specific group of experts to lead the project. The Panel identified all the procedures and treatments that constitute the cultural and clinical baggage of a Pediatrician who works in the Allergology field and entrusted them to different Committees who provided 42 specific recommendations with practical explanations, on the basis of the scientific literatures (Table 2). The results of each Commitees revision were re-analyzed by the specific group of interest and proposed to an external independent Certification Agency. All the procedures (with their scientific references or guidelines) are uploaded into the Society Web site and are available to every member who wants to implement one or more measures following the update modality of appropriateness. The Certification Agency will examine the request to certify one or all the procedures, and after a control will leave the certification that the specific procedures is performed in accordance to scientific basis. This result allow to meet the need not only of a Pediatric Allergology Center of third or second level (which find all the procedures) but also of a Pediatrician who desire to perform few procedures with a scientific basis.

**Table 2:** Recommentations on procedures and treatments provided by the Italian Society of Pediatric Allergology and Immunology – SIAIP

SRD - Scientific Reference Documents SIAIP	Health service
SRD SIAIP 001	Bronchial FeNO determination
SRD SIAIP 002	Management of severe persistent bronchial asthma
SRD SIAIP 003	Diagnosis and follow-up of atopic dermatitis
SRD SIAIP 004	Determination of oscillometric resistance (RINT)
SRD SIAIP 005	Ice cube test
SRD SIAIP 006	Spirometry with bronchodilator
SRD SIAIP 007	Spirometry with physical exertion (exercise induced bronco-constriction)
SRD SIAIP 008	Basic spirometry
SRD SIAIP 009	Nasal cytology
SRD SIAIP 010	Measurement of nasal nitric oxide (nFeNO)
SRD SIAIP 011	Nasal fibro endoscopy
SRD SIAIP 012	Rhinomanometry
SRD SIAIP 013	Diagnosis and follow-up of acute urticaria
SRD SIAIP 014	Diagnosis and follow-up of chronic urticaria
SRD SIAIP 015	Atopy patch test
SRD SIAIP 016	Intradermal autologous serum test
SRD SIAIP 017	In vivo diagnostic test for latex allergy: glove use test, glove rubbing test
SRD SIAIP 018	Desensitization to drugs
SRD SIAIP 019	Prick test, intradermo, patch test in the diagnosis for drug hypersensivity
SRD SIAIP 020	Drug provocation test
SRD SIAIP 021	Tolerance test for local anesthetics
SRD SIAIP 022	Compulsory and optional vaccination in a protected environment of patients with possible serious and immediate reactions
SRD SIAIP 023	Re-vaccination in a protected environment of patients with previous serious and immediate reactions to the vaccine
SRD SIAIP 024	Oral desensitization to food
SRD SIAIP 025	Oral provocation test for food additives
SRD SIAIP 026	Oral provocation test for foods
SRD SIAIP 027	Intradermal reaction in the diagnosis of allergy to hymenoptera venom
SRD SIAIP 028	Prick + prick with foods
SRD SIAIP 029	Skin prick test for foods
SRD SIAIP 030	Skin prick test for inhalants
SRD SIAIP 031	Skin prick test for hymenoptera venom
SRD SIAIP 032	Immunotherapy (SLIT or SC) for hymenoptera venom
SRD SIAIP 033	Subcataneous immunotherapy for hymenoptera venom (VIT)
SRD SIAIP 034	Prescription for immunotherapy for hymenoptera venom (VIT)
SRD SIAIP 035	Prescription of immunotherapy products with or without official authorization (AIFA), according to recent guidelines and regardless of the refundability status of the medication by the Italian national health service
SRD SIAIP 036	Latex immunotherapy
SRD SIAIP 037	Prescription of immunotherapy (SLIT) in subjects with latex allergy
Others	
SRD SIAIP 038	Advice on environmental prevention
SRD SIAIP 039	Advice for patients with allergy to mites
SRD SIAIP 040	Advice for patients with allergy to mold
SRD SIAIP 041	Pollen and mold calendar and documentation of the most relevant and common allergy-provoking species
SRD SIAIP 042	Monitoring of allergic patients using “allergymonitor”

The availability of all the procedures useful to a Pediatric Allergist should improve in any case the modality of health care: if the certification will be accepted as mean to identify who should perform in the better way, we think that the entire health care in this field should show a global better appropriateness. In addition, families and children require guidance on managing potentially long-lasting allergic disorders, such as asthma, rhinitis, atopic dermatitis, food allergy; balancing therapies against social and emotional restrictions. The recent Decree of the Italian Health Ministry on appropriateness of prescriptions [10] created several problems by eliminating the possibility for Physicians and Family Pediatricians to prescribe allergy tests. Given the huge demand for these investigations, the identification of the Pediatricians or Physician who are able to perform these procedures after receiving a scientific certification could be a proper choice, by leaving at the same time the most complex investigations to the second and third level centers. The Health Care Ministry could define a map of the centers and of the Family Pediatricians who demand the allergologic procedures with the awareness that tests and treatments will be performed with the same appropriateness and scientific basis, than reducing the unnecessary health cost.

## Conclusions

The Choosing wisely campaign have been widely used and disclosed in order to ensure the appropriateness of care. In the field of allergy, the Italian Society of Pediatric Allergology and Immunology- SIAIP has even been working to make recommendations and optimize the use of health care resources. Education is also important. Factors associated with greater knowledge are a prior practical demonstration, consultation with a pediatric allergy specialist and independently seeking additional informations from a patient organization. In practice, if this ambitious plan proposed by SIAIP will have success, the “choosing wisely” campaign might turn in the “spending wisely” campaign.
